# HIDmap: a physiology-based map of human immune system development to support animal-free research and regulatory innovation

**DOI:** 10.3389/fimmu.2026.1741650

**Published:** 2026-07-09

**Authors:** Christiane Spruck, Luiz Ladeira, Eliska Kuchovska, Marek Ostaszewski, Emilia Rita de Caro, Emanuela Corsini, Liesbet Geris, Bernard Staumont, Susann Fayyaz, Qiang Li, Fabian Grimm, Ellen Fritsche, Katharina Koch, Julia Tigges

**Affiliations:** 1 IUF-Leibniz Research Institute for Environmental Medicine, Duesseldorf, Germany; 2 Biomechanics Research Unit, GIGA Institute, University of Liège, Liège, Belgium; 3 Luxembourg Centre for Systems Biomedicine, University of Luxembourg, Esch-sur-Alzette, Luxembourg; 4 Department of Pharmacological and Biomolecular Sciences ‘Rodolfo Paoletti’, Università degli Studi di Milano, Milan, Italy; 5 Skeletal Biology and Engineering Research Center, Katholieke Universiteit (KU) Leuven, Leuven, Belgium; 6 Biomechanics Section, Department of Mechanical Engineering, Katholieke Universiteit (KU) Leuven, Leuven, Belgium; 7 Clariant Produkte (Deutschland) GmbH, Frankfurt am Main, Germany; 8 DNTOX GmbH, Duesseldorf, Germany; 9 SCAHT - Swiss Centre for Applied Human Toxicology, Basel, Switzerland; 10 Department of Pharmaceutical Sciences, University of Basel, Basel, Switzerland

**Keywords:** adverse outcome pathway (AOP), developmental immunotoxicity (DIT), human immune system development, new approach methodologies (NAMs), physiological map (PM), systems biology

## Abstract

Human immune system development is established through a sequence of tightly regulated processes occurring across multiple embryonic and fetal organs. Despite extensive research, our mechanistic understanding still relies largely on animal models, which often lack translational precision for human biology. This gap is particularly relevant for developmental immunotoxicity (DIT), where early-life exposure to environmental contaminants such as dioxins, PCBs, and PFAS has been associated with long-term immune dysfunction, including reduced vaccine responsiveness in children. To address this need, we created the Human Immune System Development Map (HIDmap), a curated, findability, accessibility, interoperability, and reusability (FAIR)-compliant digital resource that systematically captures prenatal immune ontogeny. The HIDmap integrates literature-derived data into a modular, three-layered structure linking anatomical niches, cell migration, and differentiation trajectories across 24 organs. Implemented in the MINERVA (Molecular Interaction NEtwoRks VisuAlization) platform, the HIDmap allows interactive exploration of developmental processes and molecular markers relevant to immune function. By embedding the HIDmap within the Adverse Outcome Pathway (AOP) framework, we constructed a putative AOP to demonstrate how the map can help to connect molecular initiating events related to environmental exposures - such as Aryl hydrocarbon receptor (AhR) activation - to downstream immunological key events and adverse outcomes. This resource offers a human-centered foundation for curating and visualizing human developmental immunobiology and may support the future development of predictive *in vitro* New Approach Methodologies (NAMs). In the future, the HIDmap may contribute to the identification of critical windows of susceptibility and support the advancement of mechanism-based, animal-free approaches in developmental immunotoxicology.

## 1 Introduction

The immune system is a complex, distributed network of cells, tissues, and organs that orchestrates the host defense against infections, diseases, and exogenous noxae (2). Multiple organs - including the yolk sac, aorta-gonad-mesonephros (AGM) region, fetal liver, thymus, spleen, and bone marrow - play essential roles in the prenatal development of immune functions (3, 4). Human immune system development unfolds in spatially and temporally coordinated waves. Primitive hematopoiesis begins in the yolk sac around gestational week (GW) 2–3 with the emergence of early hematopoietic cells (5, 6). From GW 4–5 onward, definitive hematopoietic stem cells (HSCs) and multipotent progenitors (MPP) arise in hemogenic regions such as the AGM, marking the onset of the pro-definitive hematopoietic wave (7). These HSCs subsequently migrate to the fetal liver, where they expand and drive hepatic growth (3–5, 7–10), before colonizing the bone marrow, which remains the primary hematopoietic site throughout life (5–10).

To date, most mechanistic insights into immune ontogeny stem from animal models. However, species-specific differences, ethical concerns, and limited translational relevance highlight the need for human-based New Approach Methodologies (NAMs), including *in vitro* and *in silico* systems (11–13). This need is particularly pressing in the context of developmental immunotoxicity (DIT), where current regulatory testing strategies are limited to *in vivo* rodent studies that lack mechanistic resolution. Identifying and protecting critical windows of susceptibility during immune system development is essential to prevent long-term immuno-developmental disorders caused by environmental toxicants. Epidemiological studies demonstrate that environmental toxicants such as dioxins [e.g., 2,3,7,8-Tetrachlordibenzodioxin (TCDD)], polychlorinated biphenyls (PCBs), and per- and polyfluoroalkyl substances (PFAS) can impair immune system development in humans. Transcriptomic analyses of umbilical cord blood have linked maternal PCB and TCDD exposure to altered gene expression in immune cells and sex-specific epigenetic changes, correlating with reduced vaccine responses in male children at three years of age (14). Moreover, an assessment by the European Food Safety Authority (EFSA), combining human *in vitro* assays and *in silico* models, demonstrated immunotoxic activity of four PFAS (PFOA, PFOS, PFNA, PFHxS) at human-relevant exposure levels, mirroring epidemiological findings of reduced vaccine efficacy (15–19). These findings highlight the need for human-relevant DIT testing strategies to effectively assess the developmental immunotoxic risks of environmental toxicants.

DIT has recently been identified as a Key Area of Regulatory Challenge (KARC) by the European Chemicals Agency (ECHA) in its KARC 2023, 2024, and 2025 documents (20–22), underscoring its relevance for future risk assessment and the transition toward human-relevant safety testing frameworks. Despite this recognition, uncertainties regarding the critical windows of immune system development and associated mechanisms of developmental immunotoxicants remain a prerequisite to the development of targeted testing strategies for DIT using NAMs, particularly in regulatory contexts (23). Such approaches could facilitate the reduction and replacement of animal testing requirements, including in the context of current regulatory information requirements, i.e., DIT cohorts in Extended One-Generation Reproductive Toxicity studies (EOGRTS; OECD Test Guideline (TG) 443), in favor of human-relevant and resource-efficient alternatives. These mechanistic gaps can be addressed by integrating experimental data into Adverse Outcome Pathways (AOPs) (24, 25). At present, there is no single dedicated DIT-specific AOP, although lessons from the field of developmental neurotoxicity (DNT) demonstrate how mechanistic knowledge gaps can be addressed using AOPs and NAMs. First, DNT AOPs (26, 27) link standard approaches and NAMs and support mechanism-based next generation risk assessment (NGRA) (28), particularly when embedded in integrated approaches to testing and assessment (IATA) (29). Second, the establishment of the DNT *in vitro* battery (DNT IVB) provides a regulatory-relevant example of how key developmental processes can be mechanistically mapped and translated into NAM-based risk assessment (15, 30–35). A similar conceptual framework could accelerate the development of predictive, standardized approaches for DIT.

To achieve this, a structured, physiology-based overview of prenatal immune system development is essential. Physiological maps (PM) provide standardized, expert-curated models of biological processes and offer an integrative framework for mechanistic understanding across disciplines (36–40). They can serve as a reference for basic immunology, computational modeling, test method and AOP development and further toxicological applications (36).

Here, we present the Human Immune System Development Map (HIDmap; https://disease-maps.io/immunedev/) offering a visual modular and computational representation of prenatal human immune system development relevant for research and regulatory applications.

## 2 Materials and methods

### 2.1 Literature search and selection

The initial foundation of the HIDmap was established using nine studies selected based on expert judgement and their relevance to human immune system development (7, 41–48). To expand the HIDmap and address remaining gaps, a structured literature search was conducted in PubMed on September 23, 2022, using the following query:

(Immune system AND development AND hematopoiesis) NOT (cancer) NOT (gut) NOT (breast feeding) NOT (atherosclerosis) NOT (covid-19) NOT (brain).

Only publications from the preceding five years (September 2017 to September 2022) were considered. This timeframe was selected to prioritize contemporary high-resolution datasets relevant to human immune development while maintaining a manageable scope for the initial HIDmap version. The search yielded 284 publications, which were imported to Sysrev, an online platform supporting systematic literature review through customizable labeling and filtering criteria (49).

**Figure 1:**
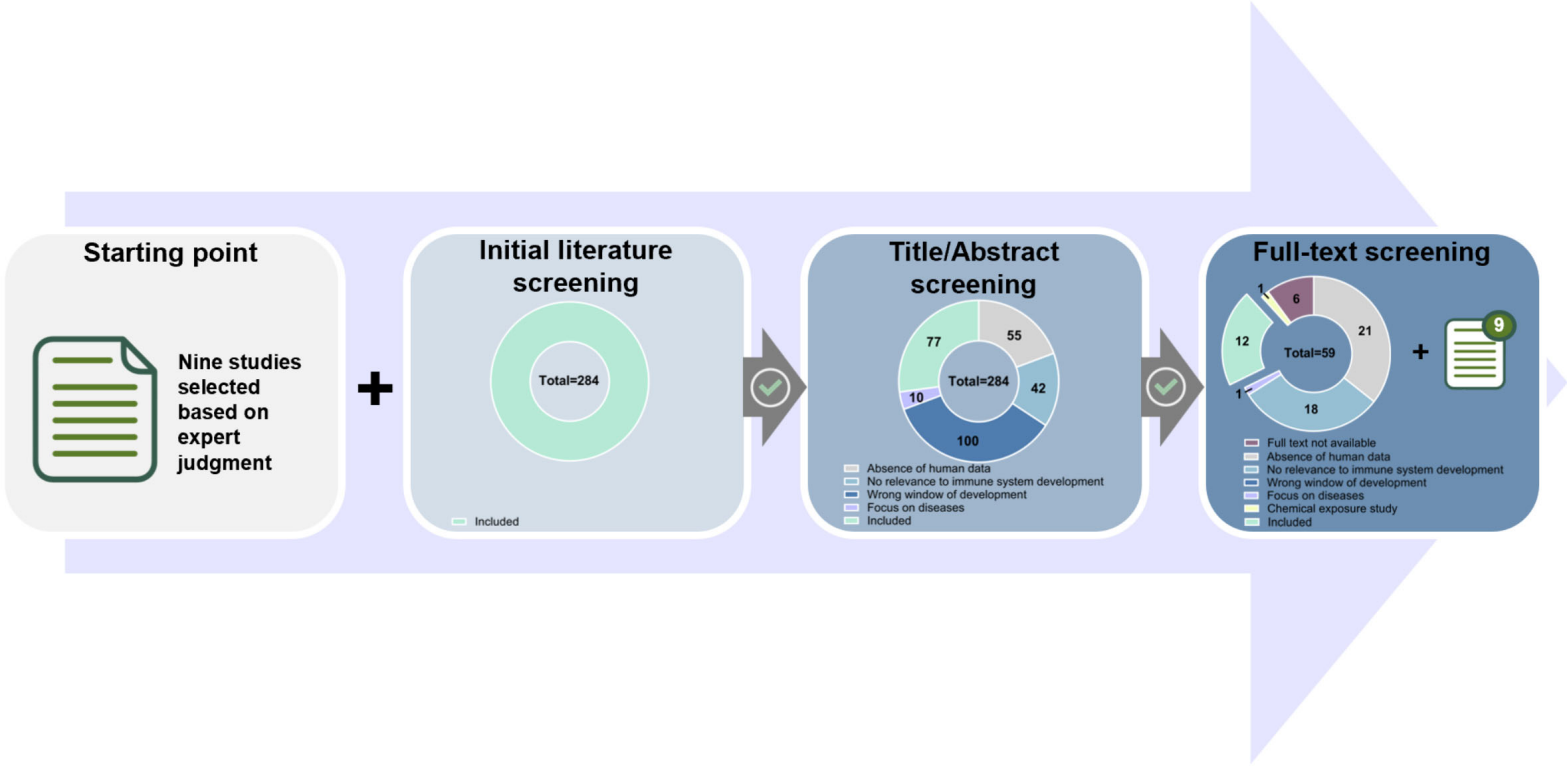
Workflow for literature screening with Sysrev to identify studies on human immune system development. The process began with the manual inclusion of nine studies based on expert judgment. An additional 284 studies were identified through a PubMed search (as of September 23, 2022) and subjected to three sequential screening steps: initial literature screening, title/abstract screening, and full-text screening. Following title and abstract review, 59 studies were retained. Of these, 12 met the inclusion criteria after full-text evaluation. Studies were excluded at each stage based on the following criteria: no relevance to immune system development, focus on disease outcomes, chemical exposure studies, or lack of human data. Donut charts indicate the number of included (green) and excluded studies at each step.

A two-step screening process was applied: (i) title and abstract screening using the exclusion criteria “absence of human data”, “no relevance to immune system development”, “wrong window of development” (excluding postnatal data), and “focus on diseases”, and (ii) full-text screening applying the same criteria together with the additional criterion: “full text not available”. Following this process, twelve additional publications were identified as primary sources for HIDmap construction (50–61). The full selection process is outlined in **Figure 1**. The HIDmap is based exclusively on peer-reviewed publications. In total, 21 publications were included, of which 10 used single- cell RNA-seq data (42, 46, 47, 50, 54, 56, 58–61), thereby providing a high level of molecular detail. Multiple data modalities were integrated into the HIDmap, including single-cell datasets, histological findings, and flow cytometry data. At this exploratory stage of HIDmap development, no formal evidence-weighting strategy was applied across different data modalities.

### 2.2 Network curation

**Figure 2:**
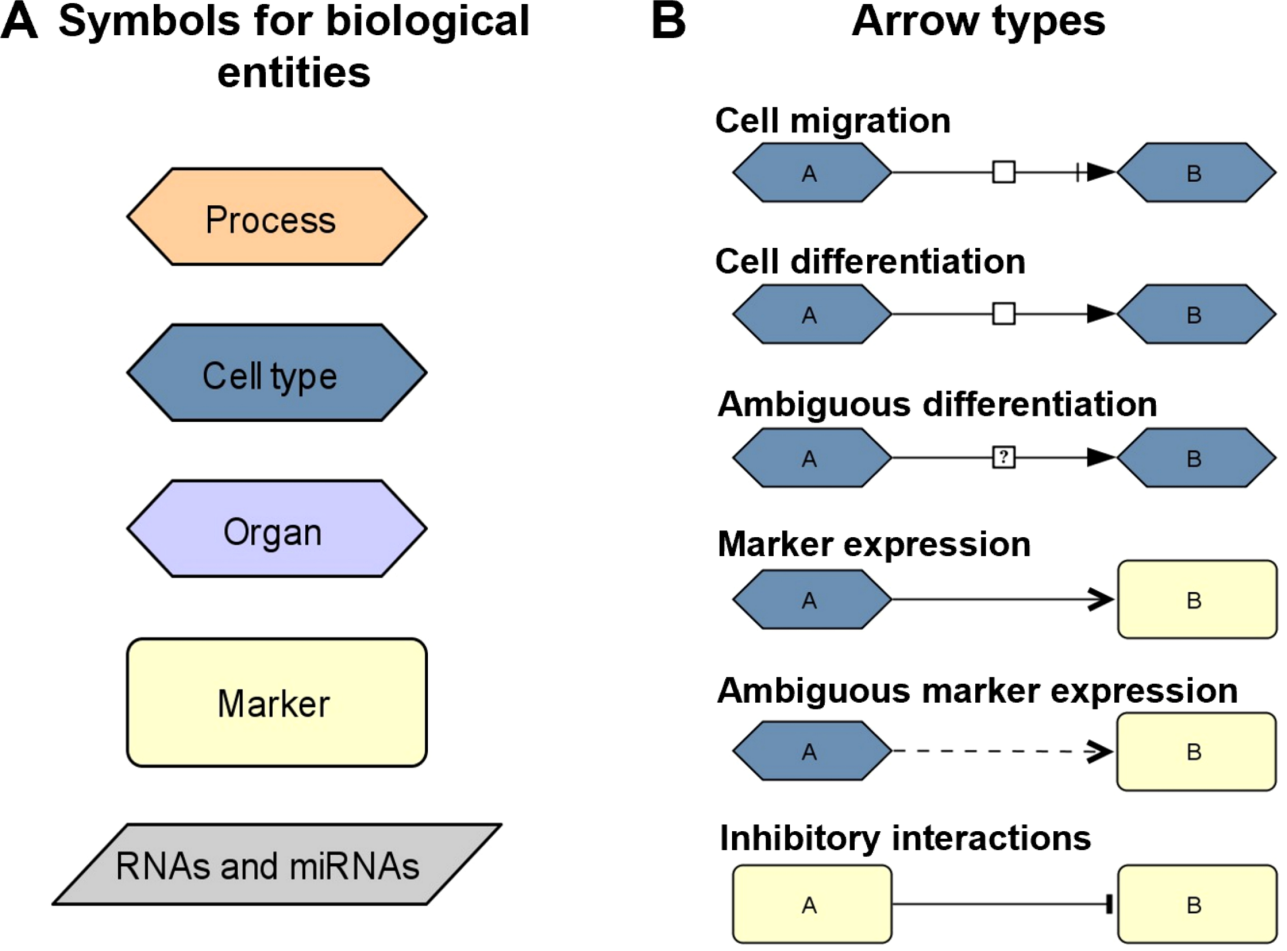
Color and symbol coding used in HIDmap created with CellDesigner. **(A)** Color scheme for different biological entities. The HIDmap was designed to be accessible to individuals with red-green color blindness: processes are shown in orange, cells types in blue, organs/locations in purple, markers in yellow, and RNAs/miRNAs in gray. **(B)** Overview of arrow types used to represent biological relationships in CellDesigner (version 4.4.2; 15, 16). Transport arrows indicate cell migration; state transition arrows indicate differentiation; positive influence arrows indicate marker expression (RNA/protein). Unknown transition and unknown positive influence arrows represent ambiguous differentiation or marker expression, respectively. Negative influence arrows indicate inhibitory interactions.

Biochemical and gene-regulatory networks for the HIDmap were constructed using CellDesigner (version 4.4.2), a process diagram editor based on the Systems Biology Markup Language (SBML) (62, 63) and the Systems Biology Graphical Notation (SBGN) standards (64, 65). The activity flow notation of SBGN was adapted (**Figure 2**) and employed, as it emphasizes biological functions while maintaining a simplified representation suitable for high-level interpretation (64–66). Each arrow was manually curated and annotated with relevant literature using PubMed Identifier (PMID), following the Minimal Information Requested in the Annotation of Models (MIRIAM) guidelines (67). All elements were encoded in a standardized format to support transparency and traceability.

### 2.3 Resource architecture

**Figure 3:**
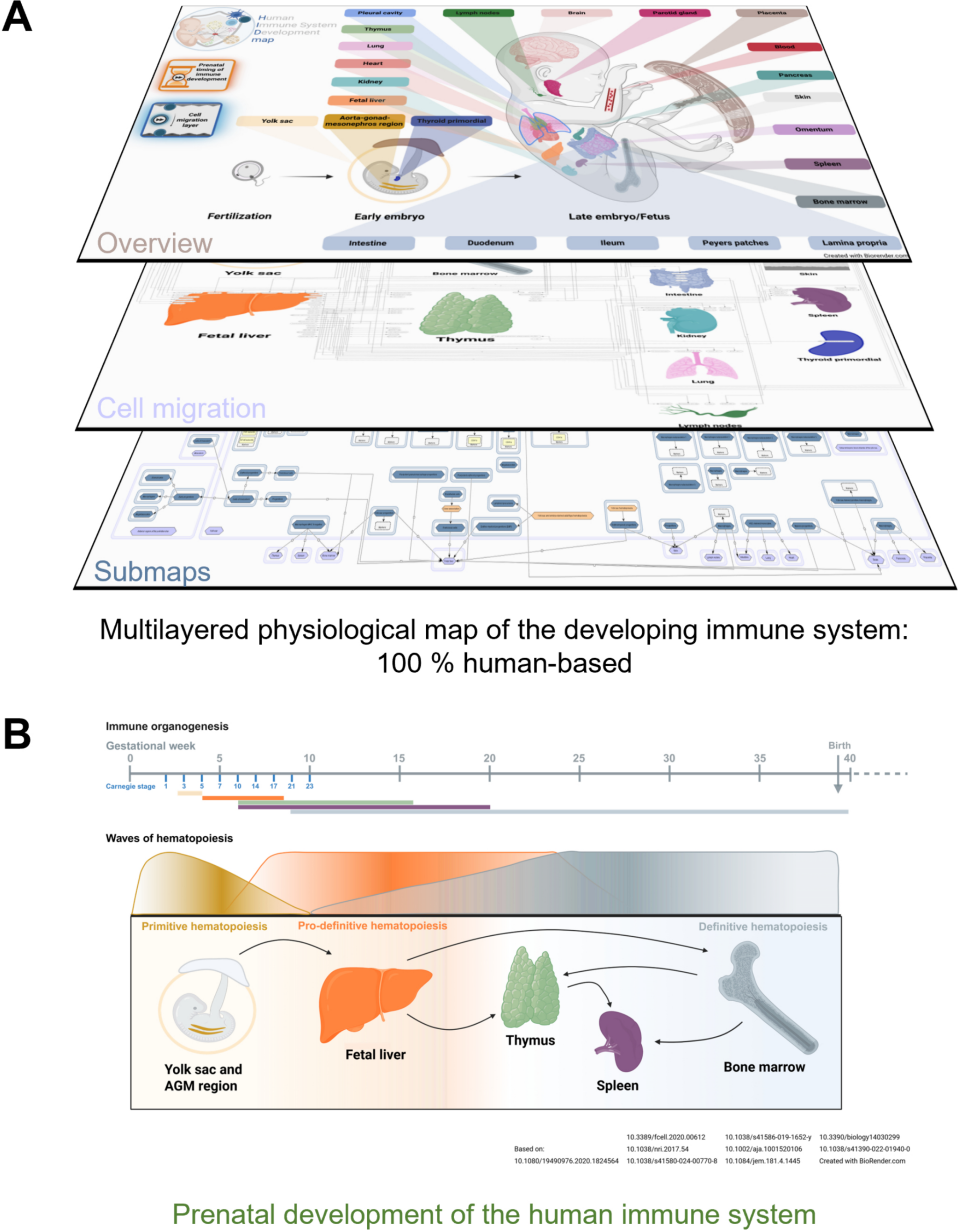
Structure of the human immune system development map (HIDmap). **(A)** The HIDmap is organized into three hierarchical levels. The first (overview) displays anatomical structures and organs involved in immune system development. The second (migration) depicts the spatial migration patterns of immune cell types between organs. The third level comprises 24 organ-specific submaps, illustrating detailed information on cell differentiation and marker expression, including protein and gene expression. The HIDmap is entirely based on human data. **(B)** The figure illustrates prenatal immune system development, showing immune organogenesis and waves of hematopoiesis. Gestational weeks and Carnegie stages are displayed as a timeline at the top. Below, organogenesis is depicted as color-coded bars (yolk sac: yellow; fetal liver: orange; thymus: green; spleen: purple; bone marrow: gray) with the three waves of hematopoiesis shown at the bottom.

The HIDmap is organized into three hierarchical layers of biological granularity (**Figure 3**): The top layer provides an anatomical overview of all included organs, summarizing the spatial contextualization of human immune system development. Clicking on a specific organ directs the user to the corresponding detailed submap. Embedded in the overview layer are two buttons: One leads to a separate figure of “prenatal timing of immune development”, and the “cell migration” button links to the second layer of the map, visualizing dynamic migration of immune cells between organs such as fetal liver, thymus, and bone marrow. Schematic illustrations of organs were created using BioRender (BioRender.com) and incorporated as background elements for anatomical orientation. This layer highlights the spatial complexity of immune cell movement. The third and most detailed layer of cell differentiation can be accessed via the overview layer and features 24 organ-specific submaps. These depict immune cell differentiation pathways and include cell type-specific expression profiles of genes and proteins (markers) as annotations. Migration destinations of individual cell types are also shown, with interactive navigation links enabling navigation between organ maps. This modular framework facilitates structured development, visualization, and iterative refinement of the HIDmap. As part of the Disease Maps Community, long-term maintenance and sustainability of the resource are supported through community engagement via the Molecular Interaction Networks Visualization (MINERVA) platform, including commenting, feedback, and review of future map updates. An informative summary of the project can be found at https://disease-maps.io/immunedev/.

### 2.4 Data visualization

The HIDmap was deployed using the MINERVA platform, a web-based platform for the curation, annotation and visualization of molecular interaction networks in Systems Biology Graphical Notation (SBGN)-compatible format (63, 68). Network files created in CellDesigner were merged and uploaded to MINERVA to enable interactive navigation, standardized visual representation, and modification of the HIDmap within a user-friendly, browser-accessible environment. A detailed user guide describing navigation and functionality within the MINERVA platform is provided as **Supplementary Material** (**Supplementary Material** “HIDmap user guide”). Additional examples and tutorials are available through the Inflammatory Skin Disease map (69), https://disease-maps.io/isdguide/, and the EU Horizon Europe 2020 “Ontology-driven and artificial intelligence-based repeated dose toxicity testing of chemicals for next generation risk assessment (ONTOX)” project (37) resources (https://ontox-project.eu/physiological-maps-2/).

To support accessibility, a color scheme optimized for red-green color vision deficiency was applied (e.g., processes: orange; cell types: blue; markers: yellow; **Figure 2A**). Additionally, CellDesigner’s arrow types were adapted to distinguish different biological relationships within the HIDmap (**Figure 2B**), including transport arrows for cell migration, state transition arrows for differentiation, positive influence arrows for marker expression, unknown transition arrows for ambiguous differentiation processes, unknown positive influence arrows for ambiguous differentiation or expression, and negative influence arrows for inhibitory interactions. The nomenclature has been standardized using symbols approved by the Human Genome Organization (HUGO) Gene Nomenclature Committee (HGNC) (70).

### 2.5 Development of a putative DIT AOP

A putative AOP centered on aryl hydrocarbon receptor (AhR) activation was developed to conceptualize potential mechanisms of DIT. The AOP was informed by available scientific evidence and expert biological knowledge. Although it was not derived from a formal systematic literature review or fully aligned with the OECD “Guidance Document for developing and assessing Adverse Outcome Pathways (71)”, it represents a plausible framework intended to support hypothesis generation and to demonstrate how the HIDmap can be used to identify, contextualize, and organize key events (KE) within an AOP framework.

## 3 Results and discussion

### 3.1 Overview of HIDmap architecture and content

We developed the HIDmap, a publicly available, findability, accessibility, interoperability, and reusability (FAIR)-compliant resource that systematically visualizes prenatal human immune system development in a structured and interactive format (72). The HIDmap integrates curated literature data into a repository of systems biology diagrams and depicts immune cell differentiation and migration across embryonic and fetal immune system-related organs, including the AGM region, yolk sac, fetal liver, spleen, thymus, bone marrow, and peripheral organs such as the thyroid primordial, kidney, heart, lung, pleural cavity, lymph nodes, brain, parotid gland, placenta, blood, pancreas, skin, omentum, lamina propria, Peyer’s patches, ileum, duodenum and intestine (**Figure 3**). Within and between these tissues, the HIDmap tracks key immune cell populations such as HSCs, progenitor cells, T-cells, B-cells, dendritic cells, macrophages, and monocytes.

**Figure 4:**
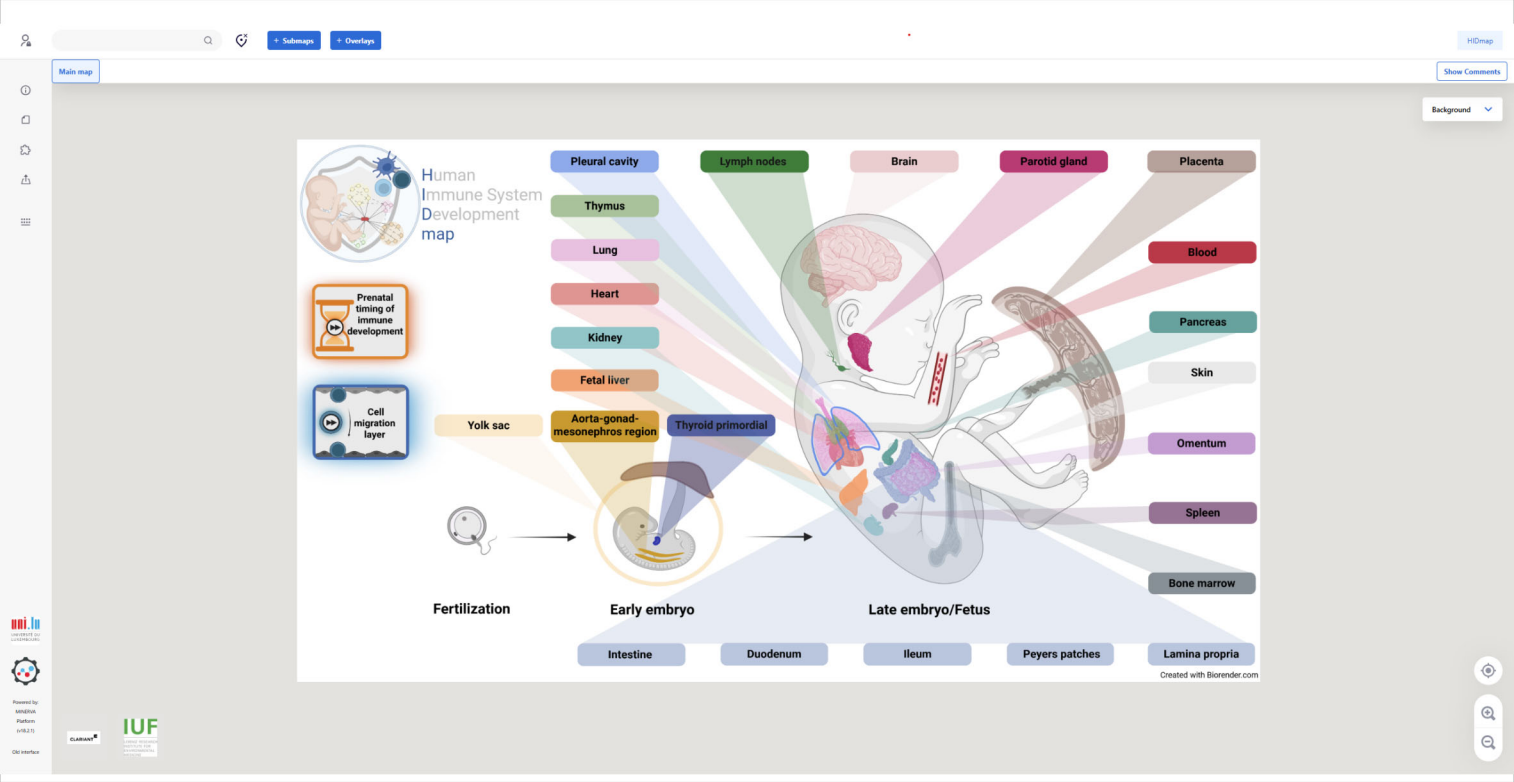
Overview layer of the HIDmap on the MINERVA platform. The overview layer illustrates the special arrangement of key organs involved in human immune system development from early embryogenesis to the fetal stage. The screenshot displays the user interface of the map on the MINERVA platform, including interactive features such as navigation tools and a search function that enables users to locate specific cell types or components of interest. The full interactive version of the map can be seen at https://immunedev.elixir-luxembourg.org/minerva/.

The HIDmap follows a three-layered architecture (**Figure 3A**) supporting spatial contextualization of immune ontogeny. The overview layer visualizes involved organs, the migration layer illustrates developmental cell migration, and the cell differentiation layer comprises 24 organ-specific submaps detailing lineage trajectories and cell type-specific marker expression profiles. In addition, a general overview of the timing is provided (**Figure 3B**). The HIDmap is publicly accessible via the MINERVA platform at https://immunedev.elixir-luxembourg.org/minerva/ (**Figure 4**).

**Figure 5:**
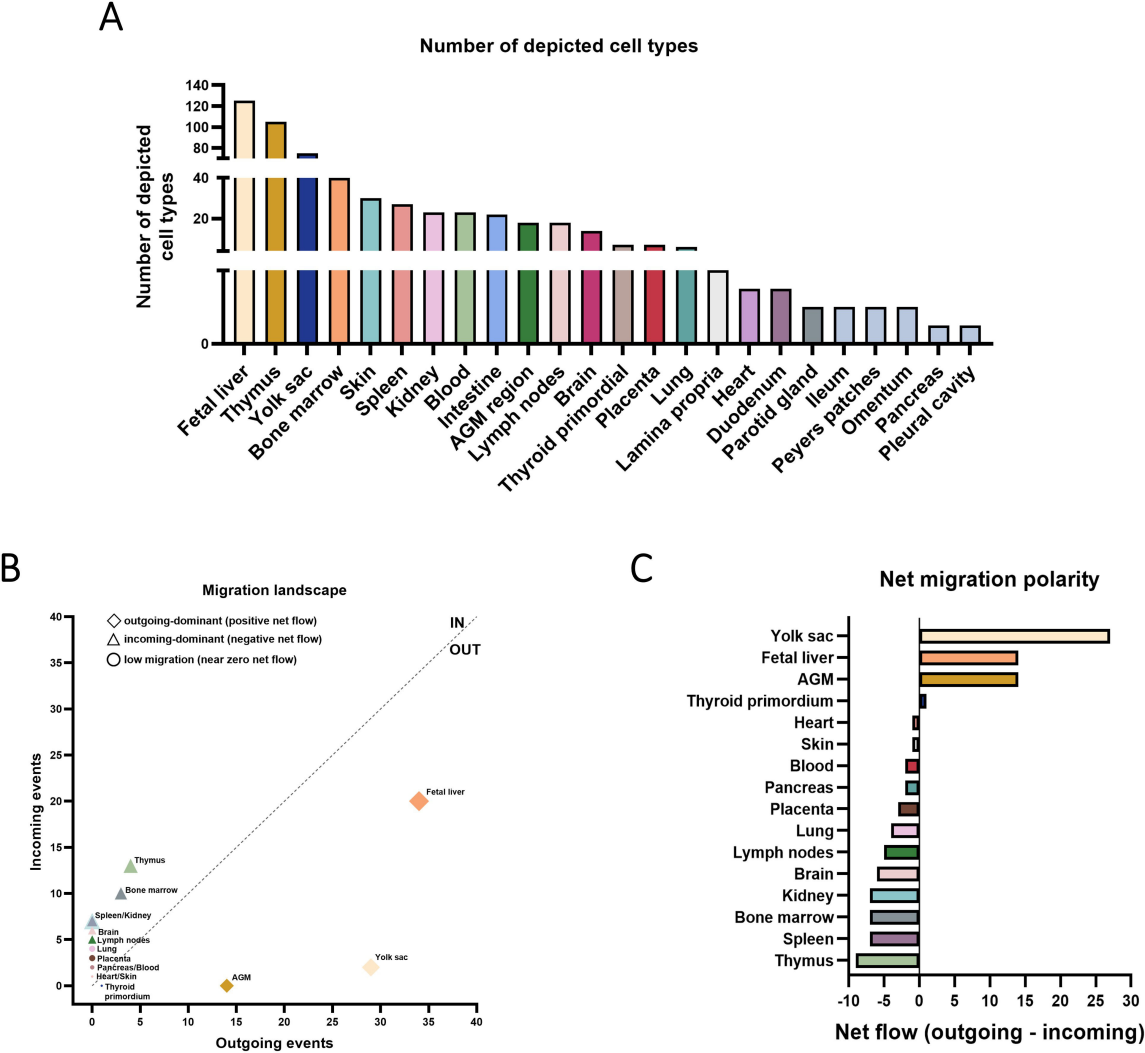
*In utero* organ coverage and migration polarity across the HIDmap. **(A)** Number of depicted immune cell types per organ across all datasets included in the HIDmap. Bars represent the total number of distinct cell types annotated for each organ. **(B)** Migration landscape showing the relationship between outgoing and incoming migratory events for each organ. Each point represents an organ, positioned according to the number of outgoing (x-axis) and incoming (y-axis) events. The dashed line indicates equal incoming and outgoing events (Out = In). Organs were categorized based on net migration bias into outgoing-dominant (positive net flow), incoming-dominant (negative net flow), or low migration (near-zero net flow). **(C)** Net migration polarity across organs, calculated as the difference between outgoing and incoming events (Out − In). Positive values indicate source-like behavior, whereas negative values indicate sink-like behavior.

**Figure 6:**
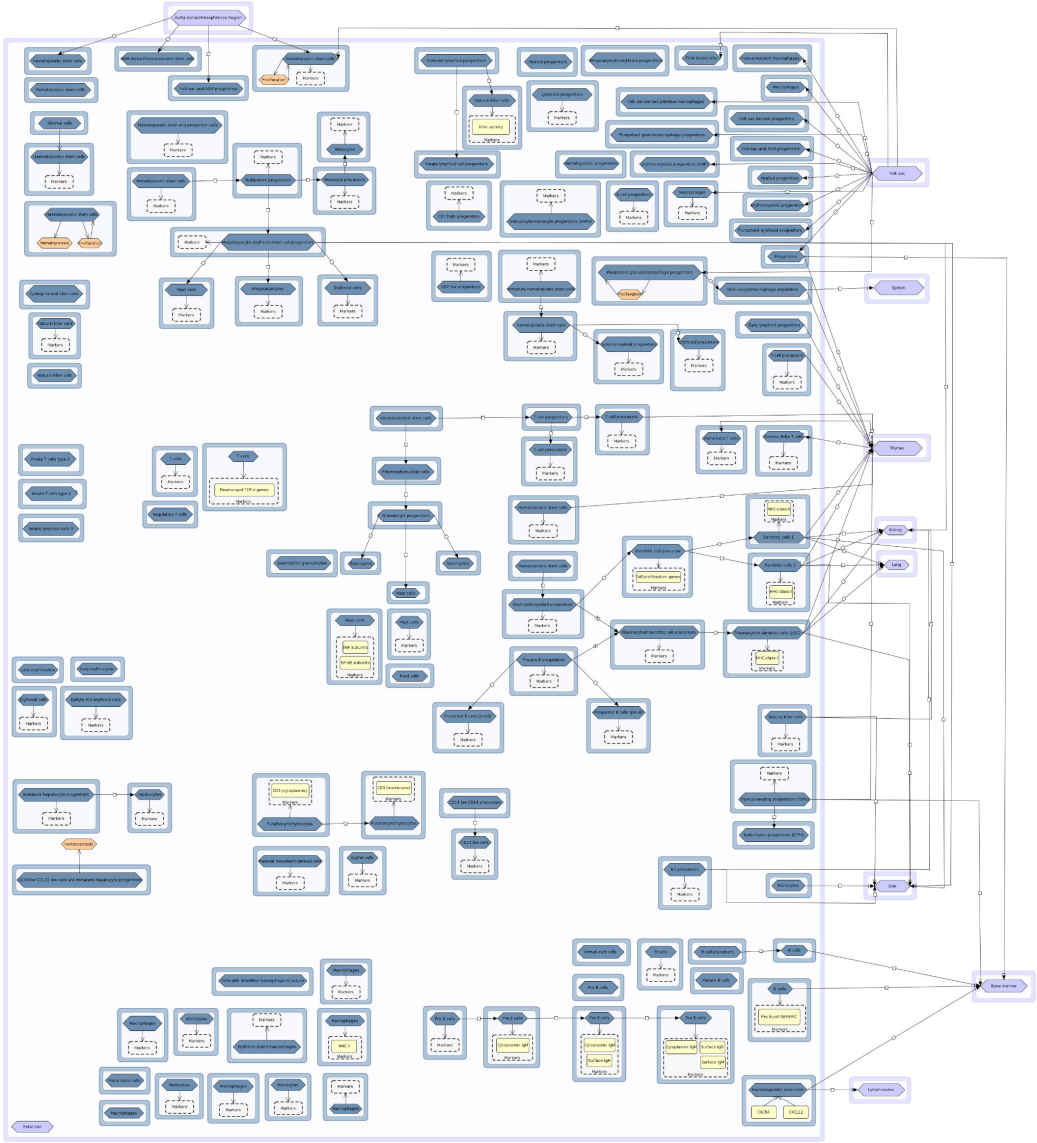
A Submap of the HIDmap illustrating the fetal liver. This organ-specific submap represents the spatial and functional organization of the fetal liver. The structure is compliant with Systems Biology Graphical Notation (SBGN) and follows the HIDmap color scheme. The organ is depicted as a bounding box enclosing an activity flow diagram. Key processes such as differentiation and migration of various immune cell types are visualized within this cellular context. The full interactive version of the map can be seen at https://immunedev.elixir-luxembourg.org/minerva/.

**Figure 7:**
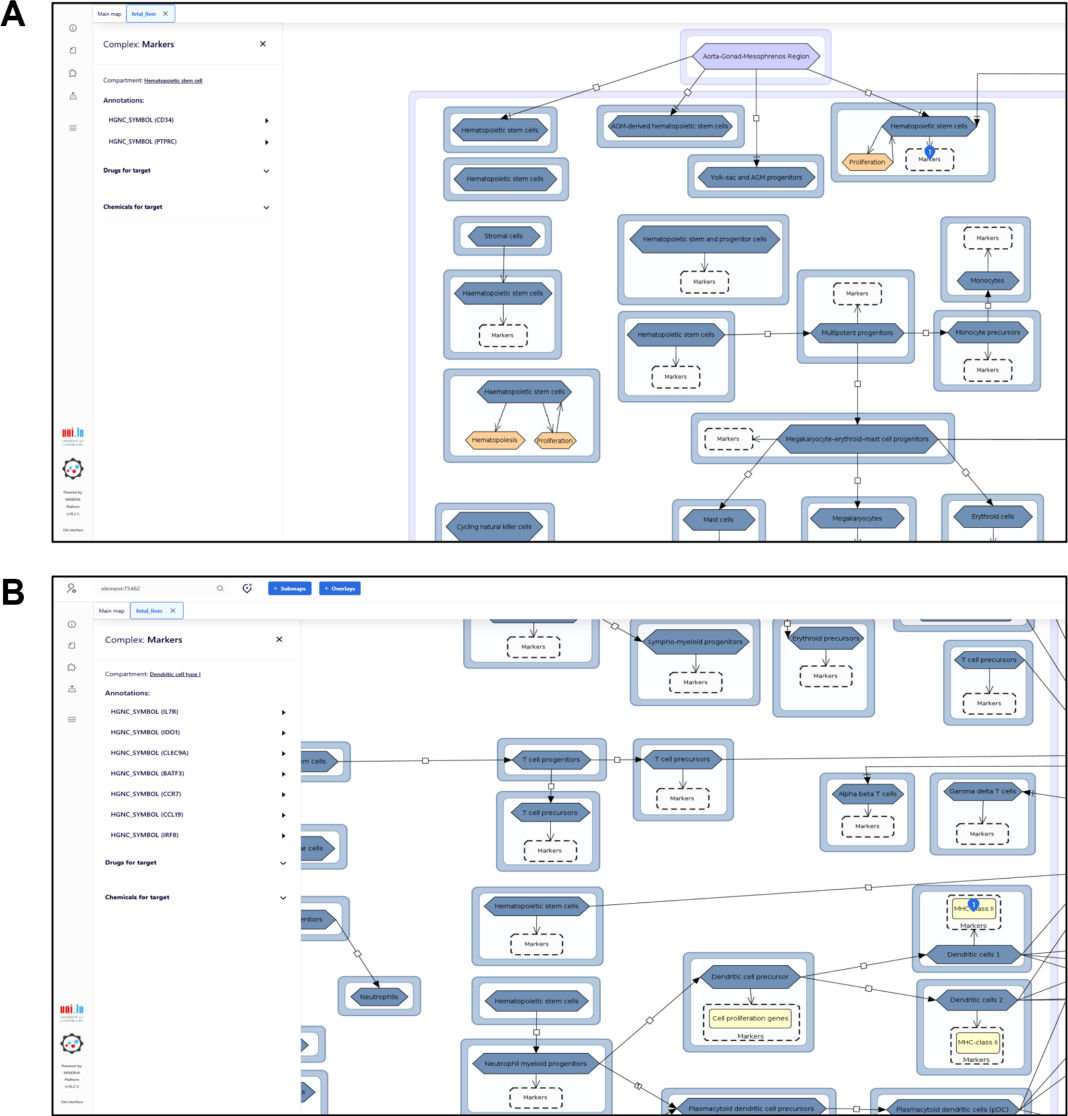
Visualization of marker expression of the different immune cells on the MINERVA platform. Standard representation of marker annotations using the official HGNC symbols are shown on the left. **(A)** Example of hematopoietic stem cell markers. **(B)** Marker annotation exceptions: yellow boxes indicate markers without an official HGNC symbol (e.g., MHC class II); this information is retained within the yellow box. Markers with official HGNC symbols remain accessible on the left after clicking on box. The full interactive version of the map can be seen at https://immunedev.elixir-luxembourg.org/minerva/.

In total, the HIDmap contains 560 cell types and 185 migration events. Annotation depth varies across 191 organs (**Figure 5A**; **Supplementary Table 1**), with the fetal liver (125 cell types), thymus (105), and the 192 yolk sac (75), representing the most comprehensively annotated developmental compartments.. By contrast, organs such as the pancreas or duodenum currently contain fewer annotated cell types (one and three, respectively), reflecting limited data availability and/or reduced representation in the literature. Migration analyses further characterized inter-organ migration relationships within the HIDmap (**Figures 5B, C**; **Supplementary Table 2**). The migration landscape analysis (**Figure 5B**) demonstrated distinct organ-specific migration profiles, with the fetal liver and AGM region showing predominantly outgoing migration patterns consistent with their roles as major developmental source compartments, whereas the thymus displayed a comparatively incoming-dominant profile. In contrast, organs such as the pancreas, thyroid primordium, and heart exhibited only a few migration events and clustered close to the origin of the migration landscape. Net migration polarity analysis (**Figure 5C**) further highlighted these differences by summarizing the balance between outgoing and incoming migration events across organs. However, these analyses reflect the current structural representation of migration relationships within the HIDmap rather than temporally resolved developmental dynamics, which remain limited by the underlying map architecture. A representative fetal liver submap is shown in **Figure 6**, illustrating cell populations and their associated marker profiles. All markers are systematically annotated using official HGNC symbols (**Figure 7A**), while non-standard markers (e.g., major histocompatibility complex (MHC) class II) are displayed in yellow boxes (**Figure 7B**) to support transparency and traceability of molecular annotations. All entries are manually curated and linked to PubMed references.

The HIDmap is designed as an interactive and continuously evolving resource. This “living” character is supported by the MINERVA Platform, which is a FAIR service (1) that enables community-driven annotation, feedback, and content refinement (68). Users can attach comments directly to specific molecular interactions or entities and upload custom data overlays to contextualize experimental datasets within the map (63). Community annotations undergo curator review before integration to maintain scientific integrity and technical standardization. In addition, MINERVA supports annotation verification against standard databases including PubMed, UniProt, and ChEBI. Tutorials and additional documentation for MINERVA are available online (https://minerva.pages.uni.lu/).

Furthermore, community-driven projects such as the Parkinson’s Disease Map (74) and the COVID-19 Disease Map (73) illustrate the utility of centralized disease maps for collaborative curation and representation of complex biological mechanisms (39).

### 3.2 Integration of the HIDmap into AOP and NAM frameworks

A central application of the HIDmap is the integration of developmental immune processes into adverse outcome pathways (AOP). AOPs provide structured representations linking molecular initiating events (MIEs), key events (KEs), and adverse outcomes (AO), whereas physiological maps (PMs) contribute mechanistic and spatial context across molecular, cellular, and tissue levels. Together, both approaches support the organization and interpretation of mechanistic knowledge relevant to DIT and NAM development. This concept has been exemplified for cytokine release syndrome (CRS), where physiological maps were used to expand AOP structures and support mechanistic interpretation and assay development (40, 75).

**Figure 8:**
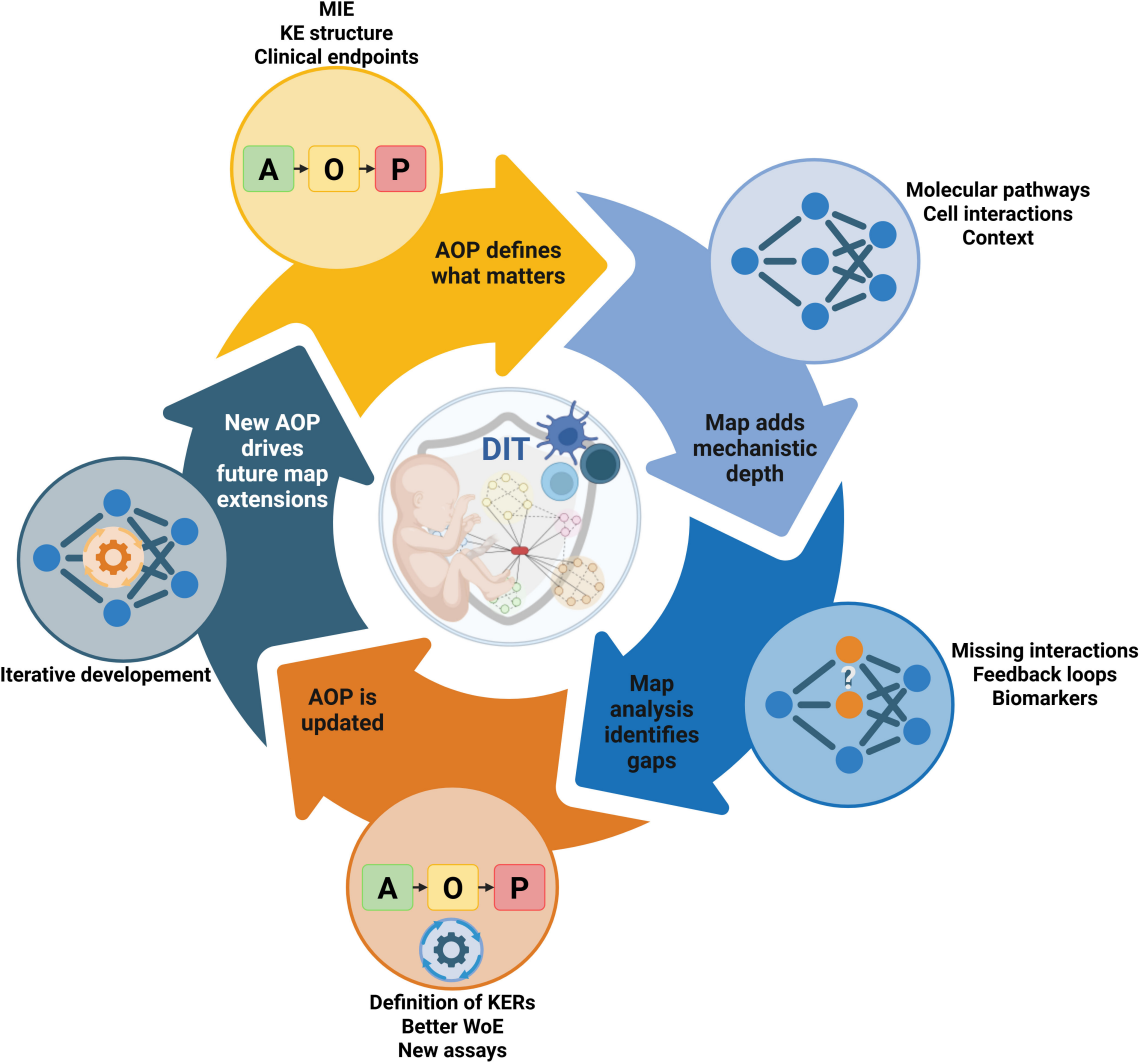
Conceptual framework for the iterative and bidirectional integration of the Human Immune System Development Map (HIDmap) and adverse outcome pathways (AOPs). The diagram illustrates how physiological maps (PMs) and AOPs work together in a complementary manner to improve our mechanistic understanding of developmental immunotoxicity (DIT). The HIDmap contributes to advancing mechanistic understanding by embedding key events (KE) in their molecular, cellular, and tissue-related context during human prenatal immune development. Subsequent analysis of the map enables the systematic identification of knowledge gaps, inconsistencies, and missing connections, which serve as the basis for refining existing KEs and adding new ones within an AOP. The revised AOP, then, guides the further expansion and curation of the PM. The updated map will then provide an enriched framework of toxicologically relevant biology for subsequent AOP iterations, thereby closing a continuous feedback loop. Through this cycle, the HIDmap and AOPs evolve together to support hypothesis-driven AOP development and to structure mechanistic representations of prenatal immune system disruptions relevant to DIT.

**Figure 9:**
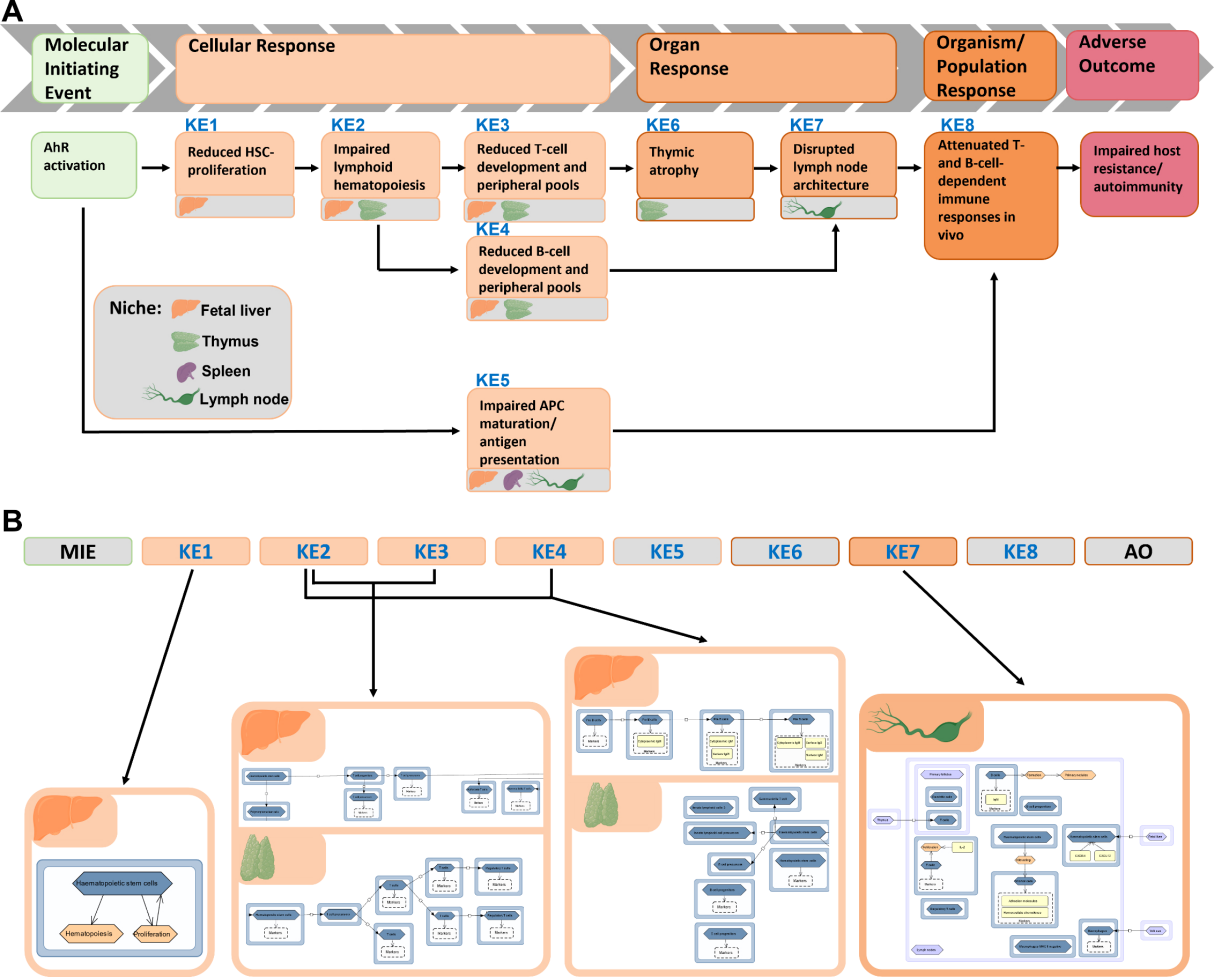
Putative AOP linking AhR activation to immunosuppression through disrupted lymphatic development and function. **(A)** Schematic representation of a putative adverse outcome pathway (AOP) describing how activation of the aryl hydrocarbon receptor (AhR) acts as a molecular initiating event (MIE), triggering a cascade of key events (KE1-KE8) that affect immune development. Early events include reduced hematopoietic stem cell proliferation (KE1) and impaired lymphatic hematopoiesis (KE2), followed by reduced T-cell (KE3) and B-cell (KE4) development and impaired maturation and antigen presentation of antigen-presenting cells (APCs, KE5). These cellular alterations manifest at the organ level as thymus atrophy (KE6) and disrupted lymph node architecture (KE7), leading to attenuated T- and B-cell-dependent immune responses that occur *in vivo* (KE8). The adverse outcome (AO) is reduced host resistance or autoimmunity. Relevant anatomical niches (fetal liver, thymus, spleen and lymph nodes) are indicated by symbols. **(B)** Integration of HIDmap elements into the putative AOP as a multi-layered visualization. Individual KEs are represented by specific HIDmap modules at the cellular or organ level, enabling structured mapping of molecular, cellular, and functional processes. To find the examples displayed, enter the following unique element numbers into the search field of the HIDmap: element:81052 (KE1); element:81139 (fetal liver), element:81780 (thymus) (KE2 + 3); element:81202 (fetal liver), element:81852 (thymus) (KE2 + 4); element:81932 (KE7).

Currently, no DIT-specific AOPs are listed in the AOP-Wiki (https://aopwiki.org/), although immunotoxicology-related examples such as AOP 154 (“Inhibition of Calcineurin Activity Leading to Impaired T-Cell Dependent Antibody Response”) (76) and AOP 277 (“Impaired IL-1R1 signaling leading to Impaired T-Cell Dependent Antibody Response”) (77) exist. Within this framework, the HIDmap may support hypothesis-driven AOP development by enabling identification and contextualization of developmentally relevant KEs within prenatal immune system development. This concept is illustrated in **Figure 8** and highlights the role of the HIDmap as a complementary framework for organizing mechanistic information related to prenatal immune system perturbations. To illustrate this concept, we developed a putative AOP involving activation of the AhR during critical windows of immune development. In this framework, AhR activation (MIE) is linked to impaired HSC proliferation (KE1) (78–80), reduced lymphoid hematopoiesis (KE2) (81), diminished T- and B-cell development and peripheral pools (KE3-KE4) (78, 81–85), and in parallel, impaired antigen-presenting cell maturation (KE5) (82, 83, 86, 87). These upstream perturbations result in thymic atrophy (KE6) (85, 88, 89), disrupted lymph node architecture (KE7) (89), and attenuated T- and B-cell dependent immune responses (KE8) (90–92), culminating in impaired host resistance and autoimmunity (90, 92) (AO; **Figure 9A**). This mechanistic cascade is supported by developmental exposure studies involving prototypical AhR ligands, including TCDD (78, 80, 81, 84, 85, 89, 91), polycyclic aromatic hydrocarbons [e.g., benzo[a]pyrene and its metabolites (88, 90, 93)] and dioxin-like PCBs (e.g., PCB 81, PCB 126) (80, 92, 94). However, this AOP has not been developed according to formal OECD guidance and should therefore be regarded as a hypothesis-generating construct rather than a validated pathway.

Within this context, the HIDmap enables spatial localization and contextualization of several upstream cellular KEs (KE1-KE4) and one KE at the organ-level (KE7) within defined developmental niches and processes (**Figure 9B**). By contrast, downstream organism-level responses (e.g., KE8 and AO) remain beyond the current scope of the HIDmap, reflecting its primary utility for upstream and cell-based key events. Thus, the HIDmap is best viewed as a structured, mechanistic framework supporting AOP development and refinement. In addition, the HIDmap may inform future NAM development by providing structured insights into developmental processes, cell migration patterns, and cell-type-specific marker expression profiles that could serve as candidate endpoints for DIT testing strategies. These curated developmental processes may guide future efforts toward the establishment of human-relevant *in vitro* test methods for assessing the DIT potential of environmental toxicants. However, additional validation, benchmarking against existing approaches, and demonstration of fitness-for-purpose will be required before regulatory application can be considered.

Lessons learned from developmental neurotoxicity (DNT) research further demonstrate how mechanistically anchored complex developmental processes can be translated into predictive *in vitro* testing strategies (30, 31, 34, 72). A similar framework for DIT, anchored in AOPs and supported by physiologically informed resources such as the HIDmap, may provide a basis for future development of predictive *in vitro* DIT testing approaches.

### 3.3 The HIDmap in the context of existing bioinformatic resources

Platforms such as Kyoto Encyclopedia of Genes and Genomes [KEGG (95),], Reactome (96), and WikiPathways (97) provide well-curated pathway representations, but do not specifically capture the context of human immune system development *in utero*. The HIDmap addresses this gap by providing an integrated, tissue-specific overview of prenatal immune system development. While these resources provide greater detail for individual pathways, such depth is beyond the scope of the current HIDmap version. Instead, the HIDmap is intended as a complementary resource providing an accessible overview of organs, cell types, markers, and key developmental processes including migration and differentiation.

Ingenuity Pathway Analysis (IPA) is a widely used commercial platform for literature-based omics interpretation and mechanistic hypothesis generation (98). However, IPA relies on generalized literature-derived networks and automated curation approaches, which may bias analyses toward well-studied pathways. In addition, IPA does not provide a developmental framework specific to prenatal immune system development and remains commercially restricted. Consequently, IPA complements rather than replaces manually curated developmental frameworks such as the HIDmap.

The Human Cell Atlas (HCA) provides high-resolution single-cell and spatial transcriptomic datasets (99), that have substantially advanced the characterization of fetal immune development and hematopoiesis. However, HCA primarily functions as a data repository rather than an integrated developmental framework and typically requires substantial bioinformatic expertise for data processing and interpretation. Consequently, HCA complements rather than replaces curated overview resources such as the HIDmap. Direct comparison of HIDmap and HCA annotations demonstrated overall concordance across major hematopoietic lineages while highlighting differences in developmental resolution. The HIDmap retained embryonic and yolk-sac-specific intermediates such as erythro-myeloid progenitors (EMP) and yolk sac-derived primitive macrophages, whereas corresponding HCA annotations were often represented by broader hematopoietic or myeloid labels. Conversely, HCA included several adult-like immune annotations that may reflect reference-atlas-driven annotation transfer rather than bona fide yolk-sac developmental states (**Supplementary Figure 1**). Notably, both resources include the yolk sac dataset published by Suo et al., 2022 (47), although the HIDmap additionally integrates information from further studies. Together, these findings suggest that the HIDmap complements HCA by preserving transient embryonic and ontogenetic information that may become obscured in broader reference-based annotation frameworks.

Taken together, these comparisons highlight the role of the HIDmap as a complementary resource integrating developmental and tissue-specific information related to prenatal immune system development. The HIDmap combines literature-derived omics, flow cytometry, and histological data into a structured and accessible framework that supports exploration of developmental markers, cell types, migration, and differentiation processes. Importantly, the HIDmap consolidates currently fragmented data on *in utero* immune system development by consolidating available knowledge onto a single platform. Unlike pathway-centered resources such as Reactome and KEGG, the HIDmap starts from developmental context and links it to relevant biological migration and differentiation processes.

### 3.4 Limitations and future directions

The current version of the HIDmap focuses on prenatal immune system development and is based on literature published between 2017 and 2022. Earlier and more recent studies are currently not represented. Future updates will incorporate newly available datasets through collaborative efforts within the EU-funded Horizon Europe project “Partnership for the Assessment of Risks from Chemicals” (PARC) (100), including the integration of literature related to monogenic immune disorders using resources from the International Union of Immunological Societies (IUIS) (101). Temporal resolution is currently limited by the underlying map architecture. Future updates will include expanded datasets and community-driven refinement of the resource. With respect to AOP development, the HIDmap primarily captures cellular processes and molecular interactions relevant to upstream and cell-based KEs. Higher-level events at the organ or organism level are only represented to a limited extent and therefore require complementary data sources.

## 4 Conclusion

In summary, the HIDmap represents a structured, human-centered framework for exploring prenatal human immune development and provides a curated digital resource integrating developmental immune processes across biological compartments during prenatal immune development. Specifically, the HIDmap addresses critical knowledge gaps identified by regulatory agencies (e.g., ECHA KARC) by providing structured, expert-curated data on immune ontogeny. Its modular, three-layered architecture enables multi-scale investigation ranging from molecular markers to organ-level processes. In addition, integration into the MINERVA platform ensures accessibility, interactivity, and community-driven sustainability. By embedding mechanistic evidence in a digital environment, the HIDmap makes biological complexity of DIT more accessible, thereby providing new opportunities for the future development of DIT-related AOPs and NAMs and offering a useful framework for mechanistic research in developmental immunotoxicology. While still at the early, exploratory stage, the HIDmap aligns with ongoing efforts to advance mechanism-based, human-relevant, and animal-free approaches to hazard identification and aligns with the 3R principles and the EU roadmap of phasing out animal experiments.

## Data Availability

The original contributions presented in the study are included in the article/**Supplementary Material**. Further inquiries can be directed to the corresponding author.

## Glossary

3R: Replacement, reduction and refinement
AGM: Aorta-gonad-mesonephros
AhR: Aryl hydrocarbon receptor
AOP: Adverse outcome pathway
BaP: Benzo[a]pyrene
CRS: Cytokine release syndrome
DIT: Developmental immunotoxicology
DNT: Developmental neurotoxicity
ECHA: European Chemicals Agency
EFSA: European Food and Safety Authority
EMP: erythro-myeloid progenitors
FAIR: Findable, accessible, interoperable, and reusable
HCA: Human Cell Atlas
HGNC: HUGO gene nomenclature committee
HIDmap: Human immune system development map
HSC: Hematopoietic stem cell
HUGO: Human Genome Organization
IGI: Inborn Errors of Immunity Committee
IPA: Ingenuity Pathway Analysis
IUIS: International Union of Immunological Societies
KARC: Key Areas of Regulatory Challenge
KE: Key event
KEGG: Kyoto Encyclopedia of Genes and Genomes
MHC: Major histocompatibility complex
MIE: Molecular initiating event
MINERVA: Molecular interaction network visualization
MIRIAM: Minimal information requested in the annotation of biochemical models
MPP: Multipotent progenitors
NAM: New approach methodology
NIPH: Norwegian Institute of Public Health
ONTOX: Ontology-driven and artificial intelligence-based repeated dose toxicity testing of chemicals for next generation risk assessment
PARC: Partnership for the Assessment of Risks from Chemicals
PCB: Polychlorinated biphenyls
PFAS: Per- and polyfluorinated alkyl substances
PM: Physiological map
PMID: PubMed identifier
SBGN: Systems biology graphical notation
SBML: Systems biology markup language
TCDD: 2,3,7,8-Tetrachlorodibenzodioxin.
