# Supplementary material for: HIDmap: a physiology-based map of human immune system development to support animal-free research and regulatory innovation
Source: Front Immunol. 2026 Jul 9;17:1741650. doi: 10.3389/fimmu.2026.1741650 (PMC13392540; doi:10.3389/fimmu.2026.1741650)
Supplement: Supplementary file 1 [file DataSheet1.zip › Data Sheet 1/HIDmap user guide.PDF]

Human Immune System Development Map (HIDmap)

https://disease-maps.io/immunedev/
Online access and exploration: https://immunedev.elixir-luxembourg.org/minerva/
Development status: Fist version is complete and published
Sustainable support: Environmental Toxicants and the Brain, MINERVA Platform
License: Creative Commons Attribution 4.0 International (CC BY 4.0) License
How to cite: https://disease-maps.io/immunedev/
Construction tool: CellDesigner
Funding: Clariant Produkte (Deutschland) GmbH, IUF - Leibniz Research Institute for Environmental Medicine
Contact: Christiane Spruck, IUF – Leibniz Research Institute for Environmental Medicine, christiane.spruck(at)iuf-duesseldorf.de

Description

The development of the human immune system is a biologically complex process involving multiple cellular niches and diverse cell types across distinct developmental stages. Current knowledge in this field largely relies on animal studies, highlighting the need for *in vitro* and *in silico* new approach methodologies (NAMs) that enable the assessment of effects on the developing immune system without animals use.

To capture the biological complexity of human immune system development and to support the design of such NAMs, we have initiated the construction of the Human Immune System Development Map (HIDmap). This physiological map (PM) – a concept derived from the Disease Maps project - focuses on the representation of normal, undisturbed physiological processes.

Based on extensive literature curation, the HIDmap provides a graphical overview of the key cellular niches and intricate biological interactions occurring during prenatal immune development, including cell migration, cell-cell interactions, and signaling molecules. The major anatomical sites involved comprise the aorta-gonad-mesonephros (AGM) region, yolk sac, fetal liver, bone marrow, spleen, and thymus. Cell types represented in the HIDmap include, but are not limited to, hematopoietic stem cells (HSCs), progenitor cells, T-cells, B-cells, dendritic cells, macrophages, and monocytes.

The main goal of the HIDmap is to comprehensively map human immune system development to identify knowledge gaps and provide a valuable reference for both research and regulatory applications in pharmacology and toxicology. In this context, the HIDmap aims to serve as a foundation for the development of adverse outcome pathways (AOPs), computational models, and human-relevant *in vitro* NAMs for toxicological testing - ultimately supporting the establishment of a NAM-based test battery for developmental immunotoxicity (DIT).

Publications

Spruck *et al*., 2026 (under review)

HIDmap User Guide

This quick guide explains how to navigate and explore the HIDmap.

Accessing the HIDmap

The main entry point is: https://immunedev.elixir-luxembourg.org/minerva/.

Upon opening, you will see an overview diagram displaying all organs included in the map, along with three buttons on the left-hand side.

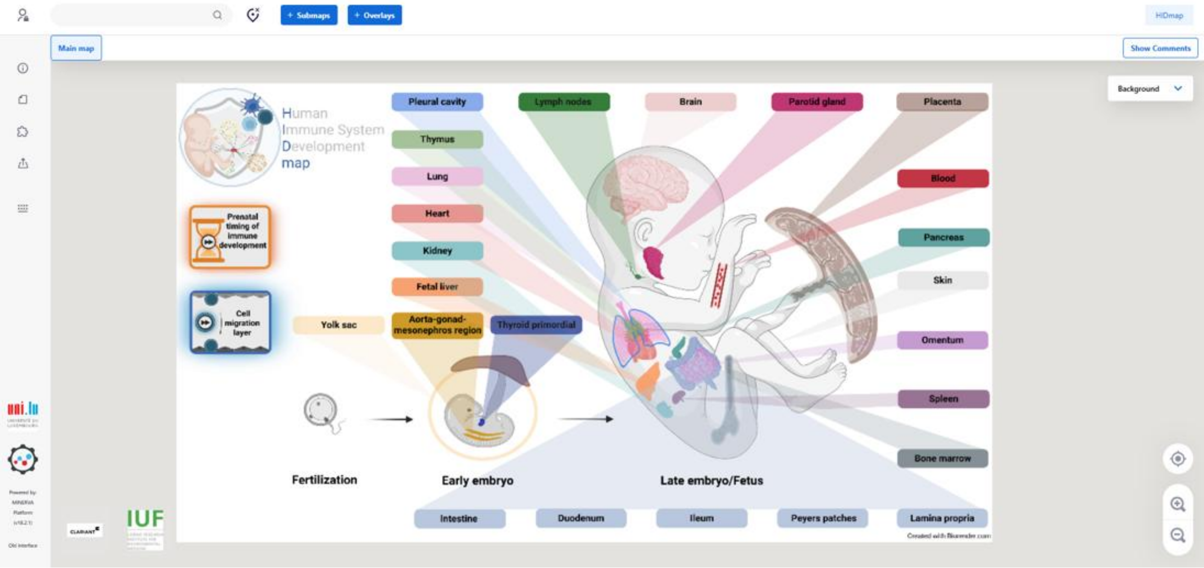

Navigating Organ Maps

To view a detailed maps for a specific organ, click on its name in the overview diagram (red arrow).

For example, selecting ‘Fetal liver’ opens the corresponding submap.

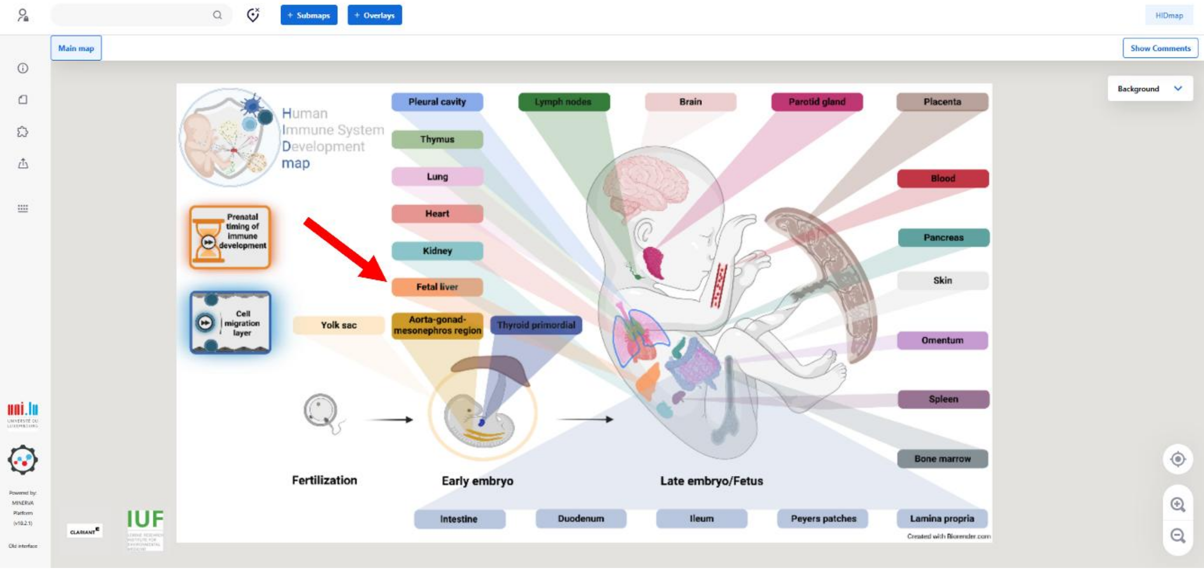

On the left-hand side, three clickable images provide shortcuts to:

- The **Disease Maps homepage** (HIDmap logo; top button; red arrow)

- The **prenatal development of the human immune system overview** (middle button; orange arrow)
- The **cells migration map**, showing inter-organ connections (bottom button; green arrow).

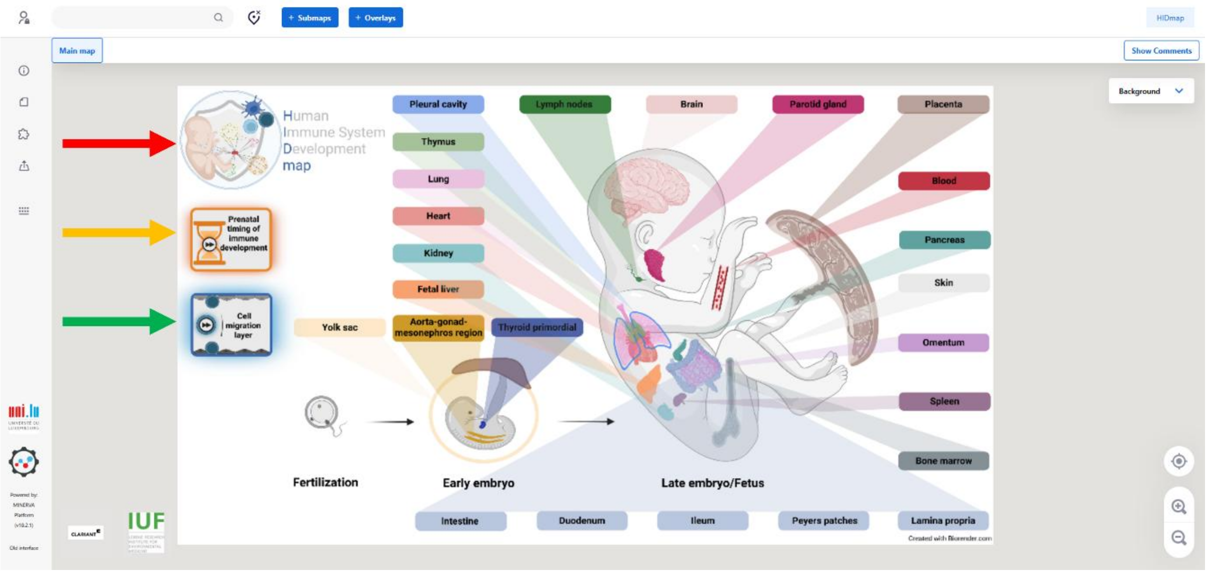

Pan and zoom

Navigation within the HIDmap works similar to Google Maps:

- Use the buttons on the **bottom right** (see the three arrows) to zoom in, zoom out, and re-center the view.
- Click and drag to move around the map.

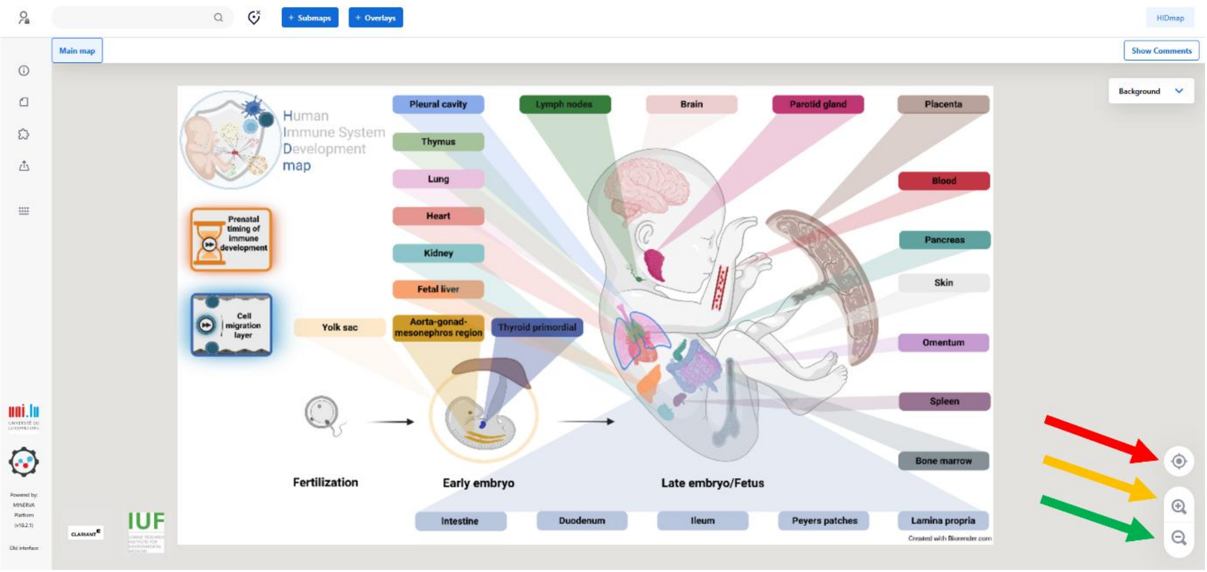

Search Function

You can search for specific elements using the **search field** (red arrow at the top left). Results are highlightedon the map with blue anchors. To clear the search, click the ‘**delete**’ button.

Search functionality is available both in the overview figure and within each submap.

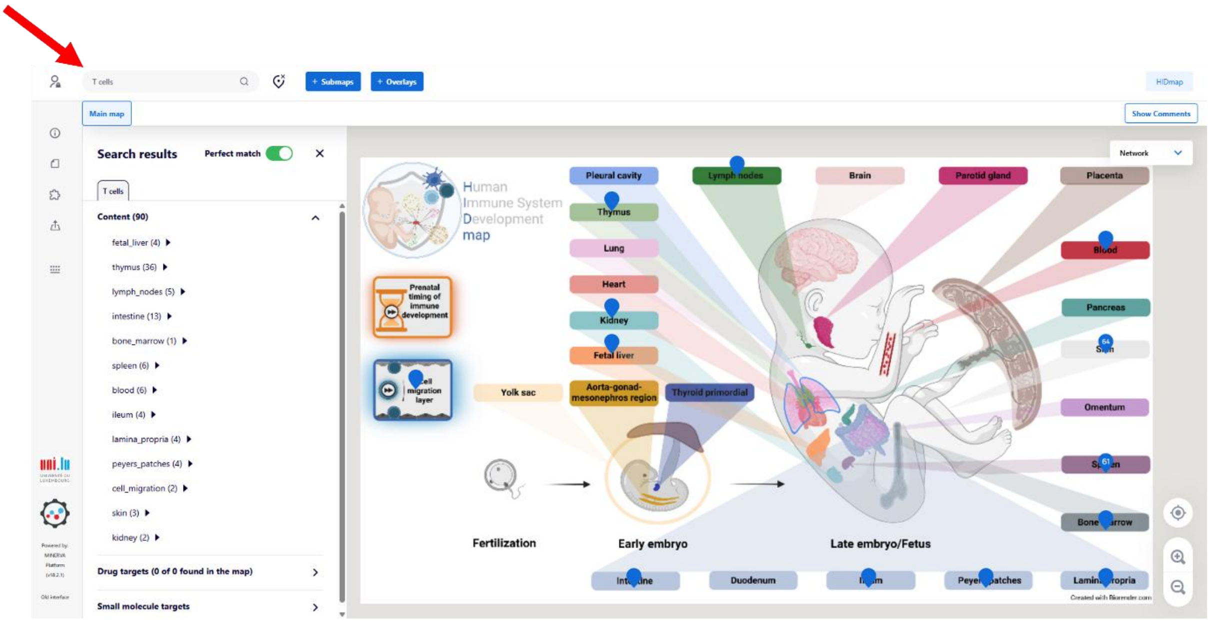

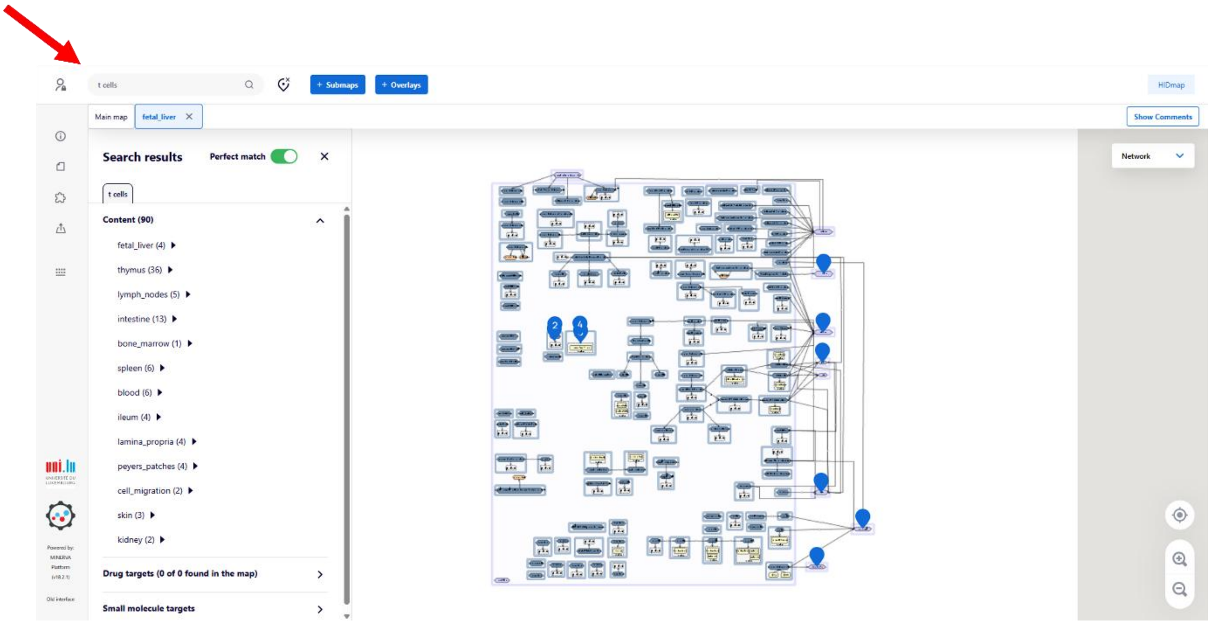

Submaps

To explore submaps, click on the '**Submaps**' button at the top (red arrow). A will appear in the left-hand panel, allowing access to:

- The main submaps (e.g. *Cell Migration* and the *Prenatal Immune Development*)
- Organ-specific submaps (e.g., *Fetal liver*, *Thymus*, *Bone Marrow*, etc.)

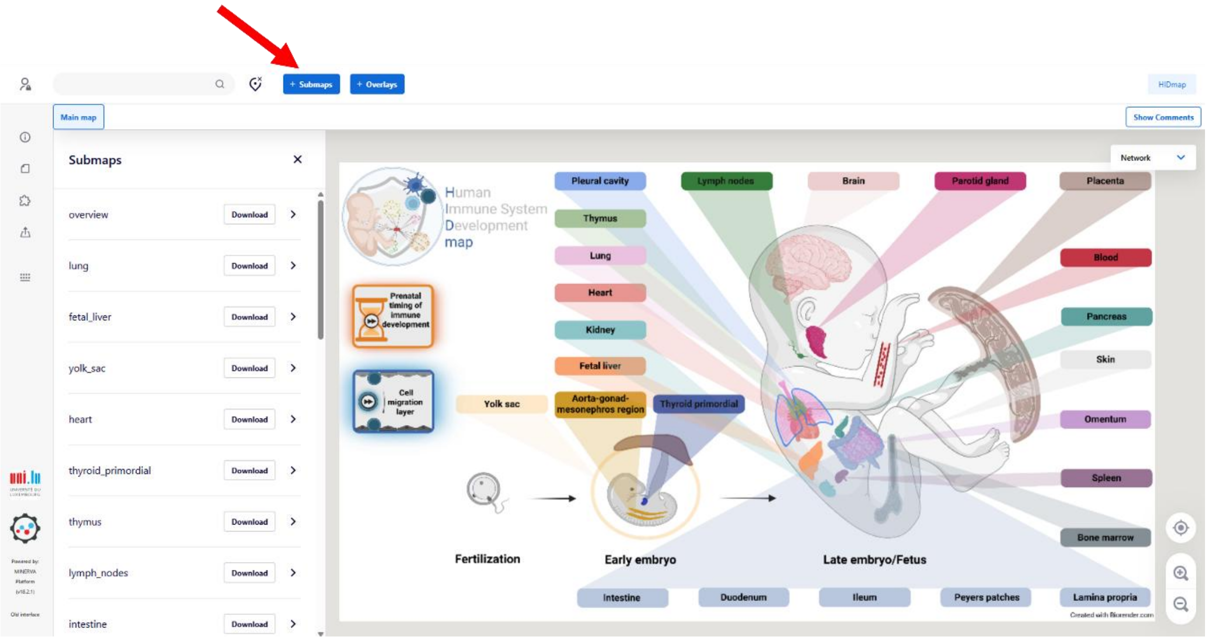

Interactive exploration

Every map element – such as **cells**, **markers**, or **interactions** - is clickable. Selecting an element opens a detailed annotation on the left-hand side, which provides:

- Curated biological information
- Official HGNC gene symbols
- Supporting PubMed references.

All molecular markers are systematically annotated with **official HGNC symbols** (A). Non-standard markers (e.g., major histocompatibility complex (MHC) class II) appearin **yellow boxes** (B), ensuring clear differentiation and traceability.

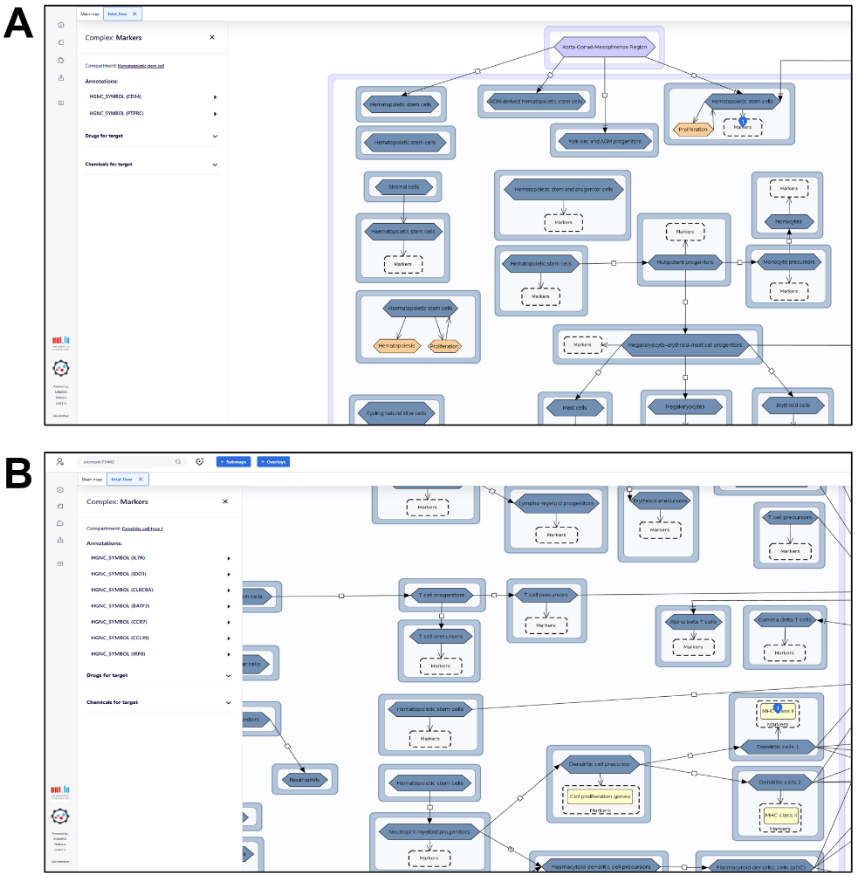

Clicking on an **interaction** (black connecting lines) displays the relevant publications supporting that biological relationship.

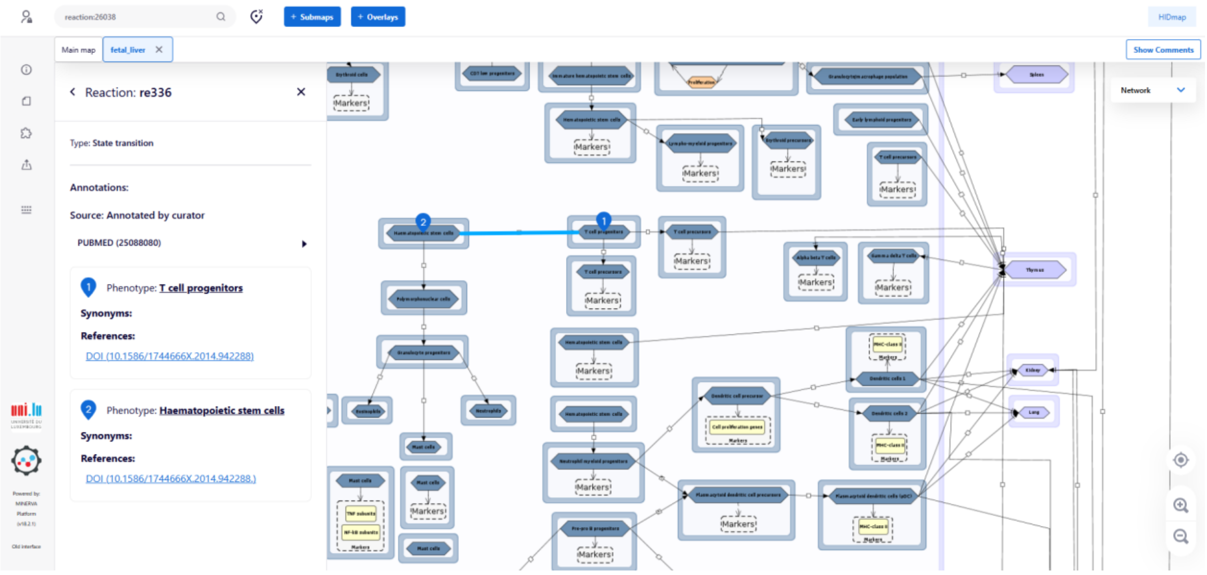

Tip

Use the combination of search, submaps, and interaction details to explore the developmental pathways and inter-organ relationships of the developing human immune system.

Development Team:

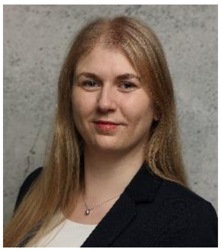	**Christiane Spruck, M.Sc.**<br>**IUF – Leibniz Research Institute for Environmental Medicine**<br>Biologist, PhD Student<br>Development of the Human Immune System Development Map
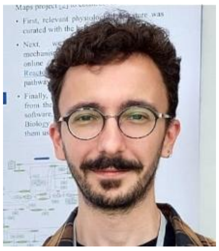	**Luiz Ladeira, PhD**<br>**GIGA Institute, Molecular & Computational Biology, University of Liège, Belgium**<br>Postdoctoral researcher<br>Curation of the Human Immune System Development Map
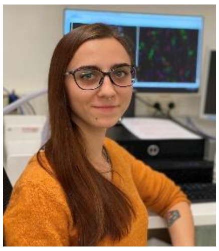	**Eliška Kuchovská, PhD**<br>**IUF – Leibniz Research Institute for Environmental Medicine**<br>Postdoctoral researcher<br>Review and editing
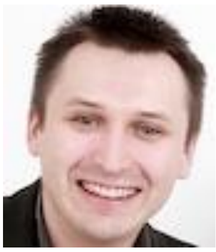	**Marek Ostaszewski, PhD**<br>**University of Luxembourg, Luxembourg**<br>Luxembourg Centre for Systems Biomedicine<br>Researcher<br>MINERVA support
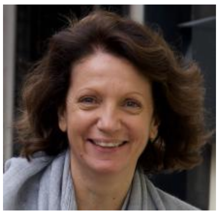	**Emanuela Corsini, PhD**<br>**DISFeB – Università degli Studi di Milano**<br>Professor of Toxicology<br>Scientific Advisor
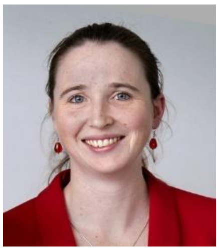	**Liesbet Geris, PhD**<br>**GIGA Institute, Molecular & Computational Biology, University of Liège, Belgium**<br>Professor in Biomechanics and Computational Tissue Engineering,<br>Supervision

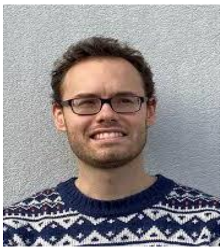	**Bernard Staumont, PhD**<br>**GIGA Institute, Molecular & Computational Biology, University of Liège, Belgium**<br>Postdoc Researcher & Project Manager, Supervision, review and editing
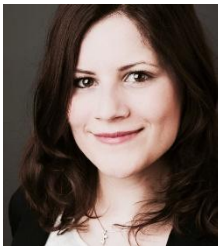	**Susann Fayyaz, PhD**<br>**Clariant Produkte (Deutschland) GmbH**<br>Expert Toxicology<br>Project administration and funding body
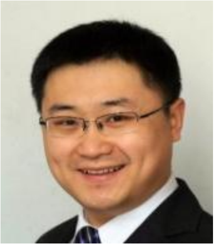	**Qiang Li, PhD**<br>**Clariant Produkte (Deutschland) GmbH**<br>**Senior Expert Toxicology**<br>Project administration and funding body
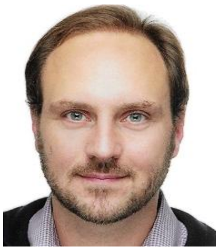	**Fabian A. Grimm, PhD**<br>**Clariant Produkte (Deutschland) GmbH**<br>Head of Toxicology and Ecotoxicology<br>Project administration and funding body
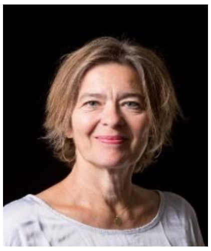	**Ellen Fritsche, MD**<br>**Swiss Centre for Applied Human Toxicology (SCAHT), Basel, Switzerland** (Director)<br>**IUF – Leibniz Research Institute for Environmental Medicine** (former working group leader)<br>Regulatory Toxicologist<br>Project generation, funding acquisition, administration and supervision
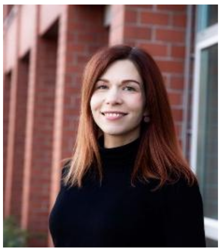	**Katharina Koch, PhD**<br>**IUF – Leibniz Research Institute for Environmental Medicine**<br>**DNTOX GmbH, Duesseldorf, Germany**<br>Scientist and junior group leader<br>Supervision, project administration and funding acquisition

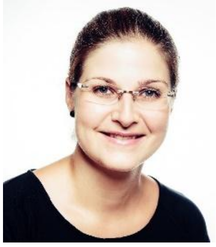	**Julia Tigges, PhD**<br>**IUF – Leibniz Research Institute for Environmental Medicine**<br>Scientist<br>Supervision, project administration and funding acquisition

Funding

- This PhD project is funded by the company Clariant Produkte (Deutschland) GmbH and the IUF - Leibniz Research Institute for Environmental Medicine.

Project progress status

★★★☆★
