# Supplementary material for: HIDmap: a physiology-based map of human immune system development to support animal-free research and regulatory innovation
Source: Front Immunol. 2026 Jul 9;17:1741650. doi: 10.3389/fimmu.2026.1741650 (PMC13392540; doi:10.3389/fimmu.2026.1741650)
Supplement: Supplementary file 1 [file DataSheet1.zip › Data Sheet 1/Supplementary figure 1.pdf]

Spruck et al., 2026
Supplementary figure 1

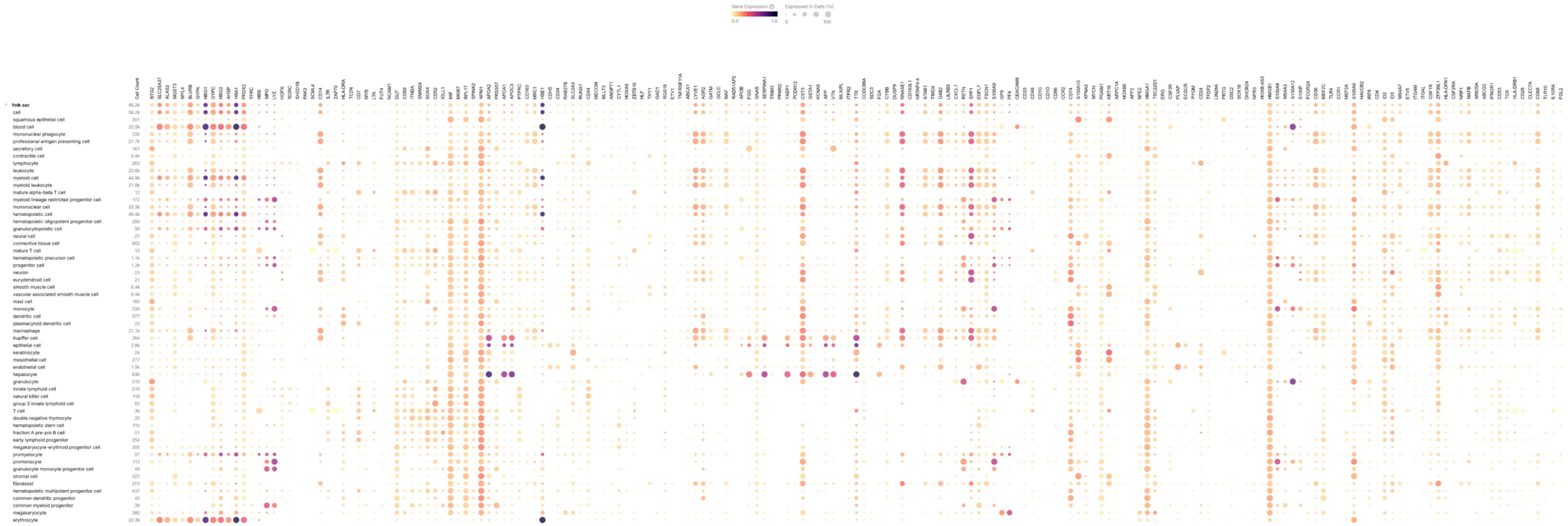

Figure S1: Cell type annotations and marker gene expression in the yolk sac of the Human Cell Atlas (HCA). Scatter plot showing the expression of curated marker genes (x-axis) across the cell types annotated by the HCA in the yolk sac (y-axis). The dot color indicates the mean scaled expression of each gene within the corresponding cell type, while the dot size reflects the percentage of cells expressing the gene (see legend at the top of the figure). The cell type annotations shown were retrieved from the CELLxGENE platform and are based on the dataset published by Suo et al., 2022.
